# Chemoenzymatic cascade depolymerization of plastics

**DOI:** 10.1038/s42004-025-01679-9

**Published:** 2025-09-09

**Authors:** Shengwei Sun, Per-Olof Syrén

**Affiliations:** 1 School of Engineering Sciences in Chemistry, Biotechnology and Health, Department of Fibre and Polymer Technology, KTH Royal Institute of Technology, Stockholm, Sweden; 2 School of Engineering Sciences in Chemistry, Biotechnology and Health, Science for Life Laboratory, Solna, Sweden

**Keywords:** Biocatalysis, Materials science, Polymer chemistry

## Abstract

Plastic waste management is challenged by the inefficiencies and environmental impact of traditional chemical recycling methods. Here, the authors explore the chemoenzymatic cascade depolymerization approach, which offers a promising and sustainable solution for transforming plastic waste into valuable products.

Since 1950, plastics have played a crucial role in modern society. Due to their numerous advantages, such as versatile physical material properties and low manufacturing costs, global plastic production reached roughly 400.3 million tons in 2022^1^ and is increasing yearly, with packaging (35%), consumer goods (23%), and construction (18%) applications accounting for the largest markets^2^. Despite its undeniable benefits, only 9% of single-use plastics are recycled, while 19% are incinerated, 50% end up in landfill and 22% evade waste management systems and go into either terrestrial or aquatic environments^3^. The massive accumulation of plastic waste in the ecosystems has posed serious threats to the various forms of life on earth. Therefore, an effective solution to tackle this crisis by transforming plastic waste into value-added products is immensely needed.

Thermal depolymerization of plastics utilizes high temperatures, whereas chemical depolymerization employs a combination of temperature control and solvents in the presence/absence of catalysts. If no catalyst is used, the depolymerization is slow, and much higher temperatures and pressures are needed^4^. Chemical recycling has gained both academic and commercial interest in the recovery of valuable building blocks from plastic waste. Apart from chemical oxidation that converts plastic into structurally modified molecules^5^, it also includes various hydrolysis reactions, such as glycolysis, microwave pyrolysis/hydrolysis, acid hydrolysis, alcoholysis, aminolysis, and catalytic pyrolysis, in which hydrolysable plastic is broken down into small oligomers or monomers^6^. The traditional chemical approaches have been considered energy-intensive and solvent-sensitive, especially to afford complete depolymerization and are projected to be able to recycle only ca 17% of all end-of-life plastics by 2060^7^. Another issue is selectivity, as often a “soup” of mixed products is observed that are difficult to separate.

In contrast, enzymatic approaches have emerged as an alternative to chemical counterparts, which offers numerous advantages such as selectivity under milder reaction conditions, reduced energy consumption, minimal environmental impact, and inherent sustainability^8^. Biocatalytic depolymerization has achieved significant advancements in the biodegradation of polyethylene terephthalate (PET) and more recently, polyurethane (PU)^9^ and polyamide (PA)^10^. Research efforts to enable depolymerization of polystyrene (PS), polyethylene (PE), polypropylene (PP), and polyvinyl chloride (PVC) are ongoing, although the biotransformations mainly rely on free-radical mechanisms that can compromise selectivity otherwise offered by an enzyme active site^11^. In particular, enzymatic depolymerization and recycling of PET into its monomers, including MHET (mono-(2-hydroxyethyl) terephthalic acid), TPA (terephthalic acid), have received much attention. Different enzymes (e.g., PETase, cutinases, esterases, lipases) have been reported to exhibit impressive degradation activity against various real-life PET materials (e.g., powders/granules, bottles, films, bags), even achieving nearly complete depolymerization at industrially relevant substrate loadings^12–15^. Yet, the efficiency of depolymerization heavily relies on the polymer’s properties, such as chemical structures, molecular weights, and the degree of crystallinity, in addition to the enzyme’s inherent activity to the particular chemical bond. For example, the FAST-PETase, a well-known variant engineered by machine learning, showed a quite low enzymatic depolymerization rate when commercial semi-crystalline PET bottles (degree of crystallinity ≥30%) were used as substrates, releasing only trace amounts of monomers (0.09 to 0.14 mM)^15^. Therefore, a more efficient method is required for plastic depolymerization.

## Chemoenzymatic cascade depolymerization of plastics

**Fig. 1:**
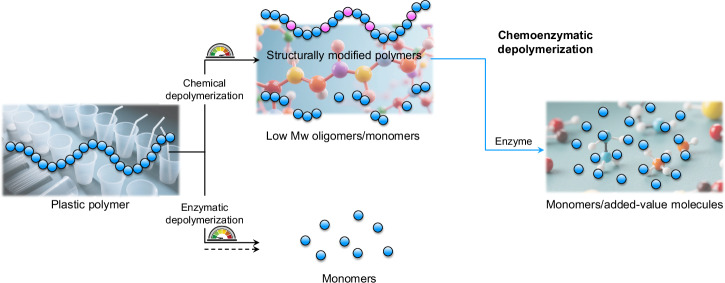
Schematic illustrations of the chemoenzymatic cascade depolymerization of plastic. For the chemical part, the plastic polymers will undergo a series of pretreatments such as hydrolysis or oxidation, producing structurally modified polymers or low *M*_w_ oligomers/monomers; for the enzymatic part, these intermediates will be further converted (e.g., hydrolyzed) into final monomers or added-value molecules in a higher yield compared to that of single enzymatic depolymerization, which might not even be working for some types of plastic (dotted line). Overall, the chemoenzymatic cascade process can accelerate plastic depolymerization, offering a more sustainable approach for plastic waste management.

Is it possible to combine chemical and enzymatic processes by chemoenzymatic cascade reactions (Fig. 1)? As a matter of fact, chemoenzymatic synthesis has been used as a multi-functional and highly sustainable synthetic tool to manufacture high-value-added organic molecules^16–18^. Likewise, there is a tremendous potential to use chemistry as a pre-treatment step to enhance material accessibility and bioactivity in enzymatic plastics deconstruction^19^. Herein, the role of the enzymes mainly is to assist in selectively when upgrading lower molecular weight molecules resulting from such chemical pretreatment^11^, such as by hydrolysis of more exposed bonds confined within the material. The final products can then be further used as feedstocks to produce new materials through chemical synthesis or implemented in metabolic engineering, achieving a circular plastics economy^20,21^.

**Fig. 2:**
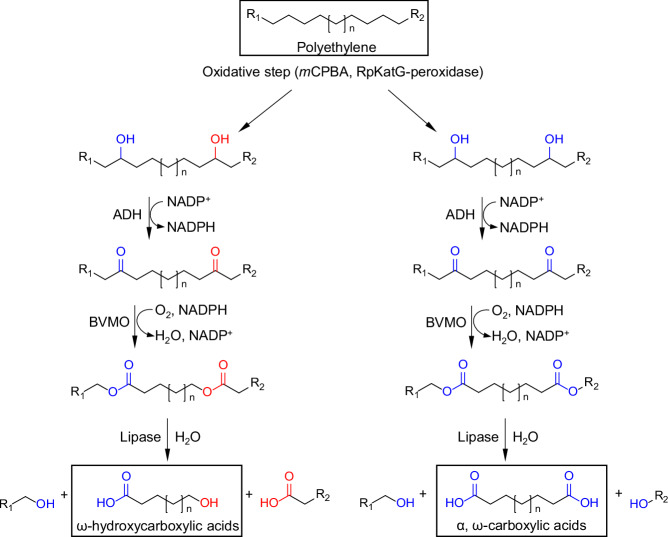
Schemes of chemoenzymatic cascade depolymerization of low-molecular-weight polyethylene. Reproduced with permission^22^. Copyright 2024, WILEY.

Bornscheuer et al. disclosed a chemoenzymatic concept for the depolymerization of low molecular weight PE^22^. The polymer was chemically pretreated with *m*-chloroperoxybenzoic acid (*m*CPBA) and ultrasonication under relatively mild temperatures (≤100 °C), followed by a four-enzyme cascade reaction, eventually leading to a ~27% polymer conversion (Fig. 2). Atomic force microscopy (AFM) confirmed the significant reduction of particle size, and gas chromatography/mass spectrometry (GC/MS) found the formation of small-molecule products including ω-hydroxycarboxylic acids and α, ω-carboxylic acids after this cascade treatment. The cleavage of *sp*^3^ C-C bonds in PE is challenging. Purely chemical depolymerization, such as noncatalytic pyrolysis and gasification, requires high-temperatures input (≥500 °C), and these processes yield uncontrolled distribution of products^23^. Incorporating versatile catalysts into the chemical depolymerization of PE does contribute to increasing the reaction efficiency at around 200–300 °C^24,25^. While efficient, a mild, simple, green, and sustainable strategy is needed for recycling of PE waste. Clearly, the work by Bornscheuer et al. represents an important step forward in the depolymerization of PE based on chemistry coupled with a biological method. However, further improvements are needed as *m*CPBA is toxic; accordingly, an alternative chemical or pretreatment route could be employed to achieve the same effects in a safer manner. The complex procedure observed and the necessity of four enzymes potentially increase the cost, time, scope, and quality if applied in industry. Moreover, it would be important to perform the Life Cycle Assessment to evaluate the environmental impacts of the entire process.

**Fig. 3:**
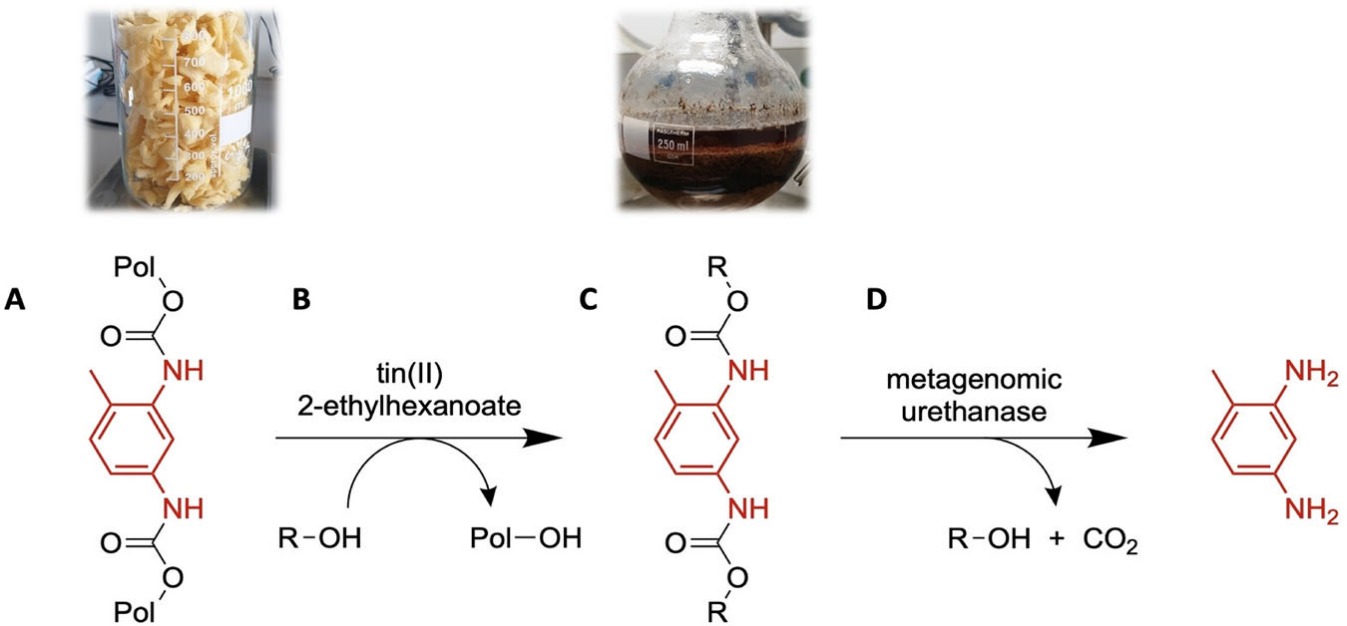
Schemes of chemoenzymatic recycling of TDI-based polyether-polyurethane foam. PU foam (**A**) as substrate and tin (II)-2-ethylhexanoate as catalyst (**B**) to generate intermediate products (**C**) subsequently converted by metagenomic urethanase (**D**) used by Bornscheuer et al. to yield final aromatic diamine (TDA). Reproduced with permission^28^. Copyright 2023, WILEY.

For PU recycling, glycolysis is one of the promising routes, as it gives a highly pure polyol moiety and under mild reaction conditions. Recent examples include deamination resulting in polyols recovered from the PU glycolysis^26^ and a glycolysis process of PU waste by Riccardo et al.^27^. In comparison, a two-step chemoenzymatic recycling procedure for PU was reported recently, which consisted of pretreatment by glycolysis followed by enzymatic hydrolysis^28^. Chemical glycolysis of polyether-PU foam resulted in the formation of low molecular weight dicarbamates. Subsequently, recently discovered urethanases identified from metagenomic analysis hydrolyzed these intermediates into the final monomer toluene-2,4-diamine (TDA) (Fig. 3). This work stands out as a new breakthrough in the depolymerization of polyether-based PU since the enzymatic hydrolysis of polyester-based PU that has been frequently reported in the previous studies rely on hydrolysis of ester bonds^29,30^. However, challenges remain in the combination of glycolysis and enzymatic degradation. It is essential to select a suitable catalyst to perform the glycolysis, which not only affects the hydrolysis efficiency but also possibly inactivates enzymes later in the process. More steps such as neutralization and separation are likely involved to ensure the compatibility between the complex chemical reaction system and the enzymatic process.

Recent years have seen tremendous progress in the chemoenzymatic cascade valorization of PET^31,32^, polyamide (PA)^33^, PS^34^, and PET/polylactic acid (PLA) blends^35^. Among these, glycolysis is generally used as a chemical pretreatment of plastic in this two-step depolymerization. The reasons could be the high efficiency, relatively mild conditions compared to many pyrolysis methods (e.g., relatively low temperature of 200 °C) and flexible choice of various catalysts. Working with highly active enzymes, this chemo-bioprocess has been suggested to be potentially applicable to the recycling and upcycling of large-scale plastic waste. However, it would be desirable to use a noncatalytic, efficient, and more environmentally friendly method to pretreat these plastics before deploying a bio-depolymerization.

**Fig. 4:**
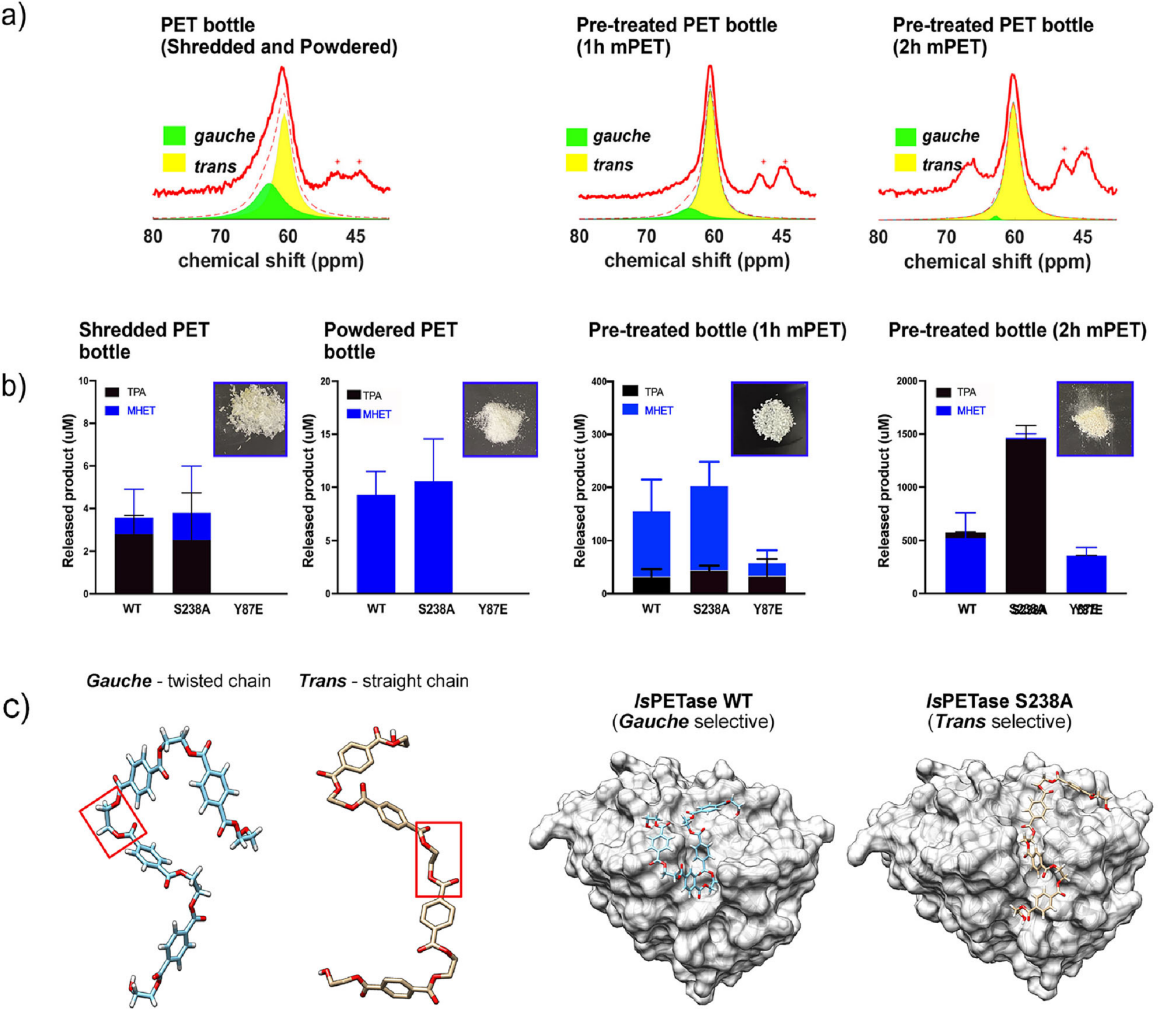
Schematic illustrations of conformational selection in PETase and S238A. **a** Distribution of conformations (*trans* vs *gauche*) for untreated and by microwave pretreated PET confirmed by solid-state NMR (**b**) and responding enhanced enzymatic degradation activity by matching enzyme and substrate conformations. **c** Scheme of conformational selection. A and B is adapted from our previous work^36^ (CC BY 4.0).

In our recent study, microwave-assisted hydrolysis (polymer in water at 200 °C for 1–2 h), followed by PETase/S238A biodegradation, has shown high potential in PET depolymerization^36^. It was found that the microwave pretreatment resulted in the polymer chain’s conformation transitioning from *gauche* to *trans* using solid-state nuclear magnetic resonance (NMR) whereas in silico simulations and atomic force microscopy (AFM) were used to confirm *trans* selectivity of S238A PETase. At this point, the *trans*-selective variant S238A exhibited higher degradation activity compared to the wild-type enzyme, in particular for commercial water bottles (Fig. 4). Our study highlighted the importance of conformational selection in biocatalytic plastic hydrolysis. It also demonstrated that microwave pretreatment (here hydrolysis instead of pyrolysis) could modify the properties of the polymer (e.g., chain conformation, molecular weight, and crystallinity) and make it more susceptible to enzymatic degradation. In principle, this combination of microwave pretreatment and enzymatic depolymerization can be extended to other polymer plastics, further accelerating enzymatic cascade depolymerization.

## Challenges for chemoenzymatic cascade reaction processes

These wide ranges of successful examples have shown how enzymatic processes potentially enhance the efficiency, sustainability and applicability of chemical depolymerization for producing various valuable products. Thus, chemoenzymatic cascade depolymerization has already offered a proof-of-concept pipeline for the recycling and upcycling of plastics. However, several challenges remain. First, besides mechanical grinding to generate particles with increased surface area, there is no general formula of chemical pretreatment on various plastic polymers. In fact, different methods, such as microwave pre-treatment introduced by us for biocatalytic plastic recycling, may bring about diverse effects or even unwanted or no actions on these macromolecules (i.e., not all molecules absorb microwave radiation). Second, for non-hydrolysable petro-polymers (PE, PP, PS and PVC) with carbon chain backbones, enzyme activities remain largely unknown. Third, the enzyme’s activity or stability can be lower than expected, due to the release of byproducts from chemical processes or additives, and the complex reaction systems (e.g., the need for various chemical catalysts in the reaction mixture, metal ions, organic solvents, extreme pH conditions, high temperatures and pressures). The often stringent requirement of controllable conditions (e.g., temperature, pH, substrate concentration) for high enzyme activity can be described as reaction incompatibility. Fourth, a green, sustainable, cost-effective, and environmentally friendly pipeline of these two-step or one-pot chemoenzymatic cascade depolymerizations remains to be demonstrated. There is often a trade-off between reaction efficiency and environmental concerns as well as economic feasibility. Fifth, the recycling of plastics into final monomers is highly desired but faces challenges such as low yield and low purity, especially when blended plastic waste is used, causing high separation costs, hindering large-scale industrial applications.

## Outlook

In the coming future, many efforts will focus on process optimizations and innovations to enable viable chemical cascade depolymerization of plastics. Integration of chromatographic and spectroscopic techniques such as high-resolution liquid chromatography coupled to tandem mass spectrometry (LC-MS/MS) will facilitate the identification of all intermediates and reaction species involved that affect biocatalyst performance in situ (e.g., various antioxidants, UV absorbers, light stabilizers and plasticizers). Moreover, a multi-enzyme cascade reaction could further increase the decomposition rate of the intermediates produced from the initial and intertwined chemical processes and allow the realization of much more complex product synthetic schemes^37^. A key priority will be the development of novel and tailor-made biocatalysts showing new-to-nature activities by de novo protein design^38–41^. Here the intrinsic stability often shown by de novo designed enzymes in concert with their unprecedented activities makes them highly suitable to be applied in chemoenzymatic cascade depolymerization strategies of difficult-to-recycle polymers. In this way, addressing chemical reactions that are currently considered too energy-intensive and environmentally unfriendly could be realized. A prominent example would be the exceptionally tough and durable polyolefins, requiring strong oxidative chemistries leading to reactive oxygen species, e.g., hydroxyl radicals. Generation of binding domains that wrap around the plastic will help in the enzymatic depolymerization, by either changing the surface property of plastic or increasing the adhesion capability of catalytic domains of enzymes; much like cellulose binding domains in nature^42^. Metabolic engineering that requires few dedicated enzymes will open new pathways for converting rich carbon sources derived from chemical cascade depolymerization to valuable products, such as bioplastics and biosurfactants^20,43^. Perhaps led by these advancements, chemoenzymatic cascade depolymerization can contribute to mitigating plastic pollution and improving the circularity in the chemical and plastic industry beyond PET.

## Supplementary information

Transparent Peer Review file
