# Supplementary material for: Chemoenzymatic cascade depolymerization of plastics
Source: Commun Chem. 2025 Sep 9;8:272. doi: 10.1038/s42004-025-01679-9 (PMC12420817; doi:10.1038/s42004-025-01679-9)
Supplement: Supplementary file 1 — Transparent Peer Review file [file 42004_2025_1679_MOESM1_ESM.pdf]

nature portfolio

Peer Review File

Chemoenzymatic cascade depolymerization of plastics

Corresponding Author: Professor Per-Olof Syren

The manuscript was considered suitable for publication without review at Communications Chemistry.

Open Access This Peer Review File is licensed under a Creative Commons Attribution 4.0 International License, which permits use, sharing, adaptation, distribution and reproduction in any medium or format, as long as you give appropriate credit to the original author(s) and the source, provide a link to the Creative Commons license, and indicate if changes were made.
In cases where reviewers are anonymous, credit should be given to 'Anonymous Referee' and the source.
The images or other third party material in this Peer Review File are included in the article's Creative Commons license, unless indicated otherwise in a credit line to the material. If material is not included in the article's Creative Commons license and your intended use is not permitted by statutory regulation or exceeds the permitted use, you will need to obtain permission directly from the copyright holder.
To view a copy of this license, visit https://creativecommons.org/licenses/by/4.0/
